# Supplementary material for: Accuracy of BIS monitoring using a novel interface device connecting conventional needle-electrodes and BIS sensors during frontal neurosurgical procedures
Source: PLoS One. 2021 Oct 21;16(10):e0258647. doi: 10.1371/journal.pone.0258647 (PMC8530286; doi:10.1371/journal.pone.0258647)
Supplement: S1 Checklist — (PDF) [file pone.0258647.s001.pdf]

Basic Statistical Reporting for Articles Published in Biomedical Journals: The “Statistical Analyses and Methods in the Published Literature” or The SAMPL Guidelines”

Thomas A. Lang[a] and **Douglas G. Altman**[b]
[a] Principal, Tom Lang Communications and Training International
[b] Director, Centre for Statistics in Medicine, Oxford University

Have they reflected that the sciences founded on observation can only be promoted by statistics? . . . If medicine had not neglected this instrument, this means of progress, it would possess a greater number of positive truths, and stand less liable to the accusation of being a science of unfixed principles, vague and conjectural.

Jean-Etienne Dominique Esquirol, an early French psychiatrist, quoted in The Lancet, 1838 [1]

Introduction

The first major study of the quality of statistical reporting in the biomedical literature was published in 1966 [2]. Since then, dozens of similar studies have been published, every one of which has found that large proportions of articles contain errors in the application, analysis, interpretation, or reporting of statistics or in the design or conduct of research. (See, for example, references 3 through 19.) Further, large proportions of these errors are serious enough to call the authors’ conclusions into question [5,18,19]. The problem is made worse by the fact that most of these studies are of the world’s leading peer-reviewed general medical and specialty journals.

Although errors have been found in more complex statistical procedures [20,21,22], paradoxically, many errors are in basic, not advanced, statistical methods [23]. Perhaps advanced methods are suggested by consulting statisticians, who then competently perform the analyses, but it is also true that authors are far more likely to use only elementary statistical methods, if they use any at all [23-26]. Still, articles with even major errors continue to pass editorial and peer review and to be published in leading journals.

The truth is that the problem of poor statistical reporting is long-standing, widespread, potentially serious, concerns mostly basic statistics, and yet is largely unsuspected by most readers of the biomedical literature [27].

More than 30 years ago, O’Fallon and colleagues recommended that “Standards governing the content and format of statistical aspects should be developed to guide authors in the preparation of manuscripts” [28]. Despite the fact that this call has since been echoed by several others (17,18,29-32), most journals have still not included in their Instructions for Authors more than a paragraph or two about reporting statistical methods [33]. However, given that many statistical errors concern basic statistics, a

Lang T, Altman D. Basic statistical reporting for articles published in clinical medical journals: the SAMPL Guidelines. In: Smart P, Maisonneuve H, Polderman A (eds). *Science Editors' Handbook,* European Association of Science Editors, 2013. This document may be reprinted without charge but must include the original citation.

comprehensive—and comprehensible—set of reporting guidelines might improve how statistical analyses are documented.

In light of the above, we present here a set of statistical reporting guidelines suitable for medical journals to include in their Instructions for Authors. These guidelines tell authors, journal editors, and reviewers how to report basic statistical methods and results. Although these guidelines are limited to the most common statistical analyses, they are nevertheless sufficient to prevent most of the reporting deficiencies routinely found in scientific articles; they may also help to prevent some reporting errors by focusing attention on key points in the analyses.

Unlike many of other guidelines, the SAMPL guidelines were not developed by a formal consensus-building process, but they do draw considerably from published guidelines [27,34-37]. In addition, a comprehensive review of the literature on statistical reporting errors reveals near universal agreement on how to report the most common methods [27].

Statistical analyses are closely related to the design and activities of the research itself. However, our guidelines do not address the issues related to the design and conduct of research. Instead, we refer readers to the EQUATOR Network website (www.equator-network.org) where guidelines for reporting specific research designs can be found. (For example, see the CONSORT [38], TREND [39], STROBE [40]) These guidelines for reporting methodologies all include items on reporting statistics, but the guidelines presented here are more specific and complement, not duplicate, those in the methodology guidelines.

We welcome feedback and anticipate the need to update this guidance in due course.

Reporting Basic Statistical Analyses and Methods in the Published Literature: The SAMPL Guidelines for Biomedical Journals

Guiding Principles for Reporting Statistical Methods and Results

Our first guiding principle for statistical reporting comes from The International Committee of Medical Journal Editors, whose Uniform Requirements for Manuscripts Submitted to Biomedical Journals include the following excellent statement about reporting statistical analyses:

> "**Describe statistical methods with enough detail to enable a knowledgeable reader with access to the original data to verify the reported results.** [Emphasis added.] When possible, quantify findings and present them with appropriate indicators of measurement error or uncertainty (such as confidence intervals). Avoid relying solely on statistical hypothesis testing, such as *P* values, which fail to convey important information about effect size. References for the design of the study and statistical methods should be to standard works when possible (with pages stated). Define statistical terms, abbreviations, and most symbols. Specify the computer software used" [33,41].

Our second guiding principle for statistical reporting is to **provide enough detail that the results can be incorporated into other analyses.** In general, this principle requires reporting the descriptive statistics from which other statistics are derived, such as the numerators and denominators of percentages, especially in risk, odds, and hazards ratios. Likewise, P values are not sufficient for re-analysis. Needed instead are descriptive statistics for the variables being compared, including sample size of the groups involved, the estimate (or "effect size") associated with the P value, and a measure of precision for the estimate, usually a 95% confidence interval.

General Principles for Reporting Statistical Methods

Preliminary analyses

n/a • Identify any statistical procedures used to modify raw data before analysis. Examples include mathematically transforming continuous measurements to make distributions closer to the normal distribution, creating ratios or other derived variables, and collapsing continuous data into categorical data or combining categories.

Primary analyses

✓ Describe the purpose of the analysis.

✓ Identify the variables used in the analysis and summarize each with descriptive statistics.

✓ When possible, identify the smallest difference considered to be clinically important.

✓ Describe fully the main methods for analyzing the primary objectives of the study.

✓ Make clear which method was used for each analysis, rather than just listing in one place all the statistical methods used.

✓ Verify that that data conformed to the assumptions of the test used to analyze them. In particular, specify that 1) skewed data were analyzed with non-parametric tests, 2) paired data were analyzed with paired tests, and 3) ✓ the underlying relationship analyzed with linear regression models was linear.

✓ Indicate whether and how any allowance or adjustments were made for multiple comparisons

(performing multiple hypothesis tests on the same data).

n/a • If relevant, report how any outlying data were treated in the analysis.

✓ Say whether tests were one- or two-tailed and justify the use of one-tailed tests.

✓ Report the alpha level (e.g., 0.05) that defines statistical significance.

✓ Name the statistical package or program used in the analysis.

Supplementary analyses

n/a • Describe methods used for any ancillary analyses, such as sensitivity analyses, imputation of missing values, or testing of assumptions underlying methods of analysis.

✓ Identify post-hoc analyses, including unplanned subgroup analyses, as exploratory.

General Principles for Reporting Statistical Results

Reporting numbers and descriptive statistics

✓ Report numbers—especially measurements—with an appropriate degree of precision. For ease of comprehension and simplicity, round to a reasonable extent. For example, mean age can often be rounded to the nearest year without compromising either the clinical or the statistical analysis. If the smallest meaningful difference on a scale is 5 points, scores can be reported as whole numbers; decimals are not necessary.

✓ Report total sample and group sizes for each analysis.

✓ Report numerators and denominators for all percentages.

✓ Summarize data that are approximately normally distributed with means and standard deviations (SD). Use the form: mean (SD), not mean ± SD.

n/a • Summarize data that are not normally distributed with medians and interpercentile ranges, ranges, or both. Report the upper and lower boundaries of interpercentile ranges and the minimum and maximum values of ranges, not just the size of the range.

✓ Do NOT use the standard error of the mean (SE) to indicate the variability of a data set. Use standard deviations, inter-percentile ranges, or ranges instead. (The SE is an inferential statistic—it is about a 68% confidence interval—not a descriptive statistic.)

✓ Display data in tables or figures. Tables present exact values, and figures provide an overall assessment of the data.[42,43]

Reporting risk, rates, and ratios

n/a • Identify the type of rate (e.g., incidence rates; survival rates), ratio (e.g., odds ratios; hazards ratios), or risk (e.g., absolute risks; relative risk differences), being reported.

✓ Identify the quantities represented in the numerator and denominator (e.g., the number of men with prostate cancer divided by the number of men in whom prostate cancer can occur).

n/a • Identify the time period over with each rate applies.

n/a • Identify any unit of population (that is, the unit multiplier: e.g., x 100; x 10,000) associated with the rate.

n/a • Consider reporting a measure of precision (a confidence interval) for estimated risks, rates, and ratios.
