# Supplementary material for: Accuracy of BIS monitoring using a novel interface device connecting conventional needle-electrodes and BIS sensors during frontal neurosurgical procedures
Source: PLoS One. 2021 Oct 21;16(10):e0258647. doi: 10.1371/journal.pone.0258647 (PMC8530286; doi:10.1371/journal.pone.0258647)
Supplement: S1 Text — (DOCX) [file pone.0258647.s003.docx]

**S1 Text. Statistical models**

Linear mixed model: dBIS vs. indBIS

The linear mixed model was used to analyze association between direct and indirect BIS values repeatedly obtained in pairs on each patient, with unequal number of measurements between patients. IndBIS measurement from patient *i* (*i*=1,…,*n*) at time *t* (*t*=1,…,*mi*) is denoted by *Y_it_*. Similary *X_it_* represents patient *i*’s dBIS measurement at time *t*. The linear mixed model is then defined as:

$$Y_{it}= \alpha+ \beta X_{it}+a_{i}+b_{i}X_{it}+\varepsilon_{it}$$

$$a_{i} \sim N\left( 0, \sigma_{a}^{2} \right), b_{i} \sim N\left( 0, \sigma_{b}^{2} \right), \varepsilon_{it} \sim N(0, \sigma_{\varepsilon}^{2})$$

where *α* = intercept; *β* = effect of dBIS; *a_i_ =* random intercept; *b_i_* = random effect of direct BIS; and *ε_it_* = normally distributed random error.

Linear mixed model: Bland-Altman analysis

The linear mixed model was used to estimate bias and 95% limits of agreement of the Bland-Altman analysis. The difference between dBIS and indBIS values (indBIS–BIS) from patient *i* (*i*=1,…,*n*) at time *t* (*t*=1,…,*mi*) is denoted by *Y_it_*. The mixed effects model is then defined as:

$$Y_{it}= \mu+ \beta t+b_{i}+ \varepsilon_{it}$$

$$b_{i} \sim N\left( 0, \sigma_{b}^{2} \right), \varepsilon_{it} \sim N(0, \sigma_{\varepsilon}^{2})$$

where *µ* = the mean bias of interest; *β* = effect of time; *b_i_ =* random effect of the individual; and *ε_i_ =* random error.

Standard error (SE) was estimated as the square root of variance of mixed random effects. The 95% agreement limits are therefore estimated as follows:

$$\overline{Y} \pm1.96*SE$$

$SE= \sqrt{\hat{\sigma}_{b}^{2}+{\hat{\sigma}_{\varepsilon}^{2}}}$
