# Supplementary material for: Accuracy of BIS monitoring using a novel interface device connecting conventional needle-electrodes and BIS sensors during frontal neurosurgical procedures
Source: PLoS One. 2021 Oct 21;16(10):e0258647. doi: 10.1371/journal.pone.0258647 (PMC8530286; doi:10.1371/journal.pone.0258647)
Supplement: S1 File — (PDF) [file pone.0258647.s004.pdf]

― 課　題　名 ―

“市販 BIS センサ貼付困難症例における針電極使用”を念頭に置いた新規 BIS モニタリング変換デバイスの有効性と安全性に関する臨床試験

久留米大学医学部　麻酔学教室

研究責任者：　原田　秀樹

作成年月日：平成 28 年 6 月 7 日

1. 標題

“市販 BIS(bispectral index)センサ貼付困難症例における針電極使用”を念頭に置いた新規 BIS モニタリング変換デバイスの有効性と安全性に関する臨床試験

2. 研究の実施体制（学内研究組織）

研究責任者：麻酔学講座　准教授　原田　秀樹

研究分担者：麻酔学講座　助教　太田　聡
麻酔学講座　助教　原　将人
麻酔学講座　助教　木村　寛子
麻酔学講座　助教　上瀧　正三郎
麻酔学講座　助教　服部　美咲
整形外科講座　講師　山田　圭
整形外科講座　助教　井手　洋平
整形外科講座　助教　溝上　健次
脳神経外科講座　講師　坂田　清彦
脳神経外科講座　助教　折戸　公彦
脳神経外科講座　助教　菊池　清志

3. 研究の背景・意義

BIS モニタリングは、意識レベルに関係する脳皮質活動をその周波数、振幅と干渉をもとに数値化したもので、麻酔鎮静度の評価に用いられるものである。術中 BIS モニタリングを行うことで、麻酔薬の投与量をより適切にすることが可能であり、過度の鎮静および浅麻酔による術中覚醒の危険性を少なくすることができると考えられており、米国では約 70%の全身麻酔症例に使用されていると言われている。

現在、前額部に専用センサを貼付することで前頭葉脳波を取得し、BIS モニタリングを施行するのが基本だが、貼付部位が術野、清潔野、術中にセンサ確認が困難な場所となりうる頭頸部脊椎手術などにおいては、適切な位置に専用センサを装着できない場合が少なからずある。その場合、装着位置を鼻尖にずらすなどして対応しているが、前額からの距離が大きくなると far field potential が大きくなり、計測脳波の SQI(signal quality index)が低下し、信頼性に乏しくなる。このような際に、長年にわたり脳波計測に臨床使用されてきた針電極を前額部に適用し、専用センサを介して BIS モニター装置に接続可能となれば臨床的意義は極めて大きい。

今回我々は既に商品化されている針電極を、市販専用センサを介して BIS モニター

に接続するデバイスを開発したため、このデバイスの有用性・安全性を確認するため本臨床研究を企図した。

デバイスは以下の通り

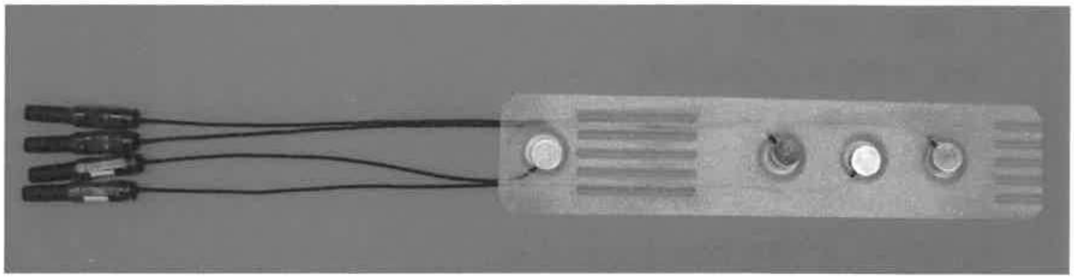

市販針電極と既存 BIS センサ間に用いる今開発した変換デバイスの全容

左の４本からなる黒い電極部分で日本光電製**脳波用針電極 NE-220B に接続可能となる。**
右側が市販 BIS センサの受け手側となる変換デバイス部分で、銀色の皿電極部分は、下記の様な試験（模式図）を経て最適な電気抵抗を取得可能とした再利用可能な銀塩化銀皿電極。

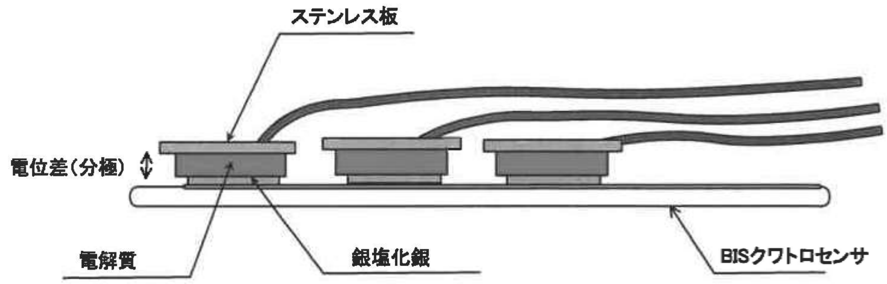

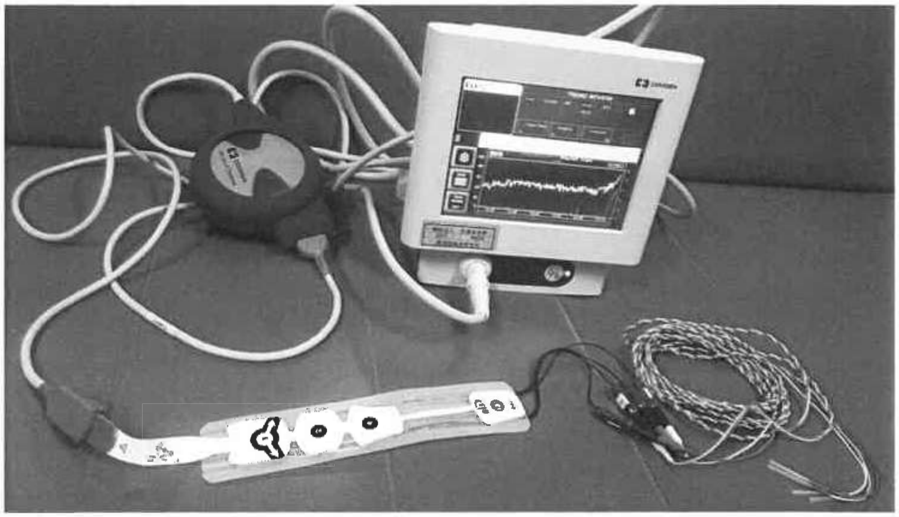

4. 研究の目的

周術期脳波モニタリング患者を対象として、既存針電極を用いた新規 BIS モニタリング変換デバイスの有効性と安全性を検討する。

5. 研究デザイン

前向き研究

6. 被験者の選定方針

6-1. 対象者および募集方法

久留米大学病院、脳外科および整形外科、全静脈麻酔にて術中管理を行う患者　目標症例数20名

6-2. 選択基準

以下の基準を全て満たす患者を対象とする

・同意取得時の満年齢が20歳以上の患者

・本人から文書による同意が得られた患者

・ASA1または2の患者

6-3. 除外基準

以下の除外基準のいずれかに該当する患者は、対象から除外する

・前額部に外傷や皮膚疾患等認める患者

・頭蓋内病変等で術前より意識レベルの異常を認める患者

・術式によりBISセンサーを適切に装着できない患者

・神経疾患を併存している患者

・精神疾患を併存している患者

・針反応陽性の患者

・金属アレルギーの患者

7. 研究期間

研究期間：平成２８年６月倫理委員会承認後から平成３０年１２月３１日まで

8. 研究方法

脳外科および整形外科の手術症例において元々電気生理学的モニタリングを施行予定の定例手術患者で本試験に対して同意を得られた患者に対して以下の手順で試験を行う。

8-1. 具体的手順

通常診療の手術室搬入後、針電極と市販 BIS センサを麻酔導入直後に被験患者の前額部に装着し、周術期意識レベルおよびBIS関連諸元値の相関を比較検討する。

Ⅰ. 導入前に針電極と市販BISセンサの両方を、前額部同じ部位に装着する

Ⅱ. プロポフォール、レミフェンタニル、ロクロニウムにより麻酔導入

Ⅲ. 通常の全身麻酔維持

Ⅳ. 針電極と市販BISセンサによる測定値は、同部位に装着するため、通常は差異を認めない。本研究で用いるBISモニターは二箇所のBIS値を表示可能であり、同部位に電極を装着し、脳波生波形の相同性を観察、処理脳波地計算値である
SQI, SEF, Beta-ratio, resting ratioなどを比較し乖離の有無・程度を記録する
Ⅴ. 手術終了後、覚醒に伴うBIS上昇における両者間の乖離の有無・程度を記録する

8-2. 使用する機器（全て臨床試用可能な医療機器）

①日本光電製脳波用針電極 NE-220B（針の長さ13mm／直径0.4mm）
②BIS™ Complete 4 Channel Monitoring System
③BIS™ Bilateral Sensor

8-3. 割り付け方法

本研究は同一患者から得られる２カ所の情報の比較であるため、割付は該当しない。

8-4. 評価項目

主要評価項目：２カ所測定部分から得られるBIS関連諸元値
副次評価項目：周術期合併症

8-5. 解析方法

Repeated ANOVA (post hoc試験)、BIS関連諸元値の経時的２群比較で、観測値が同一である事を証明する。

9. 中止基準

以下のような場合には試験を中止する
・患者から参加同意の撤回があった場合
・担当医師が試験の継続を不適当と判断した場合

10. 研究実施後の被験者への対応について

研究終了後において、当該研究の結果により得られた最善の医療（予防、診断及び治療）を受けることができるよう対応する。

11. 被験者に予期される利益と不利益

11-1. 本研究参加による期待される利益（効果）

術中の麻酔深度がより厳密に評価できる可能性はあるが、日常診療範囲内で実施される観察研究のため、研究対象者に直接の利益は生じない。研究成果により将来の医療の進歩に貢献できる可能性がある。

11-2. 本研究参加により予想される不利益・副作用・危険性

すでに長年にわたり臨床で使用されて来た日本光電製脳波用針電極 NE-220B（針の長さ13mm／直径0.4mm）を用いるため、皮下出血及び感染の可能性はあるが、偶発症は極めて少ないと考えられる。

12. 被験者の保護

12-1. 倫理原則の遵守

本研究は、ヘルシンキ宣言に基づく倫理的原則を遵守し、「人を対象とする医学系研究に関する倫理指針」に従って実施する。なお本研究を実施するにあたり、久留米大学倫理委員会にて承認を得る。

12-2. 個人情報保護の方法

研究の実施に関わる者は被験者のプライバシー及び個人情報保護に十分配慮する。研究責任者は研究の実施に際して、データ等の保護に必要な体制を整備する。研究で得られた被験者データを本研究以外の目的以外で使用する場合は、必要に応じて別途対象者から同意を得る。

13. 被験者の同意（インフォームド・コンセント）

被験者からは周術前に説明を行い同意を得る。同意撤回書も同時に手渡し麻酔導入前までに撤回書の提出、または、口頭での撤回の申し入れがあれば研究対象から除外する。

14. 被験者の費用負担、損失補償について

14-1. 費用負担

本研究は通常診療内で行われるため、健康保険の範囲内で行われ、研究期間中の観察・検査、使用薬剤等は全て患者が健康保険にて負担する。また、今回実施する針電極を用いた新規 BIS モニタリングの費用は麻酔学講座にて負担するため、患者に特別な費用負担は発生しない。

14-2. 健康被害に関する損失補償

本研究の実施に起因する健康被害が生じた場合は、その治療には原則として被験者の健康保険を使用することとし、適切な治療その他必要な措置を受けることができるように対応する。費用は患者負担にて対応するため、試験実施施設からの被験者に対する金銭等での補償は行わない。

15. 試料・情報（データ）の管理及び廃棄の方法

本研究に関するデータは、連結可能匿名化処理を行い、麻酔学講座内のインターネットに接続されていない PC 内に保管する。また、同意書等は麻酔学講座内の施錠付きロッカーに保管する。なお、保管期間は 5 年間とし、診療情報以外の研究に関する書類は全てシュレッダーにかけ処分する。

16. 本研究の資金源、利益相反について

本研究は、麻酔学講座の教室研究費にて実施するため、特定企業からの資金援助はないため、利益相反は発生しない。

17. 研究成果（特許権）の帰属先

本研究で新たな知的財産が生じた場合の権利は久留米大学に帰属する。

18. 研究結果の開示、情報公開について

本研究での研究成果は、日本麻酔科学会およびアメリカ麻酔科学会での発表及び論文により学術誌への発表を行う予定である。

19. データの二次利用について（附随研究について）

本研究で得られたデータ（試料・情報）を別の研究に利用する可能性がある。その場合の試料及び情報等は本研究と同様に匿名化し、個人情報の保護を図る。附随研究を行う場合は、改めてその研究計画を倫理委員会において審査し、承認を受けた上で利用する。

20. 問い合わせ先

久留米大学麻酔学講座　原田秀樹　医局：3606
