# Supplementary material for: Accuracy of BIS monitoring using a novel interface device connecting conventional needle-electrodes and BIS sensors during frontal neurosurgical procedures
Source: PLoS One. 2021 Oct 21;16(10):e0258647. doi: 10.1371/journal.pone.0258647 (PMC8530286; doi:10.1371/journal.pone.0258647)
Supplement: S2 File — (PDF) [file pone.0258647.s005.pdf]

— Title of the project —

A clinical study to assess clinical performance and safety of a novel interface device for BIS monitoring, which was developed for “use of needle electrodes for patients whose clinical condition does not allow conventional BIS monitoring”

Department of Anesthesiology
University of Kurume School of Medicine

Principal Investigator: Hideki Harada

Date: June 7, Heisei 28 (2016)

1. Title

A clinical study to assess clinical performance and safety of a novel interface device for BIS monitoring, which was developed to use needle electrodes for patients whose clinical condition does not allow conventional BIS monitoring

2. Organization of the investigators (intra-university research group)

Primary investigator:

Department of Anesthesiology, Associate Professor, Hideki Harada

Co-investigators:

Department of Anesthesiology, Assistant Professor, Satoshi Ota
Department of Anesthesiology, Assistant Professor, Masato Hara
Department of Anesthesiology, Assistant Professor, Hiroko Kimura
Department of Anesthesiology, Assistant Professor, Shozaburo Jotaki
Department of Anesthesiology, Assistant Professor, Misaki Hattori
Department of Plastic Surgery, Lecturer, Kei Yamada
Department of Plastic Surgery, Assistant Professor, Yohei Ide
Department of Plastic Surgery, Assistant Professor, Mizokami Kenji
Department of Neurosurgery, Lecturer, Kiyohiko Sakata
Department of Neurosurgery, Assistant Professor, Kimihiko Orito
Department of Neurosurgery, Assistant Professor, Kiyoshi Kikuchi

3. Background and rational of the study

BIS monitoring quantifies consciousness-related activity of cerebral cortex based on frequency, amplitude, and interference, and is used to assess the depth of anesthesia and sedation. Intraoperative BIS monitoring enables more appropriate dosing of anesthetics and therefore is considered to reduce the risk of intraoperative arousal due to excessive sedation or too light anesthesia. In the United States of America, the use of BIS is estimated to be about 70% of general anesthesia.

BIS monitoring is usually performed with a dedicated sensor attaching to forehead to acquire frontal lobe EEG. However, if the appropriate position of the sensor is within a surgical site or an antisepticised operative field, or if controlling the sensor by anesthetist is difficult due to cervical surgery, it is often not possible to attach a dedicated sensor to an appropriate position. In

those cases the attachment position is shifted toward nasal tip. However, if the distance is increased, the reliability of BIS becomes poor, due to an increased far field potential and a lowered signal quality index (SQI) of the electroencephalogram (EEG).

In such cases, it would be of extremely great clinical significance, if the needle electrodes, which have been clinically used for many years, are placed in forehead and can be connected to the BIS monitoring device via a dedicated sensor.

We developed a device that connects commercially available needle electrodes and the BIS monitor via a commercially available dedicated sensor for BIS. Therefore, we planned this clinical study to assess the clinical performance and safety of this device.

The device is as follows:

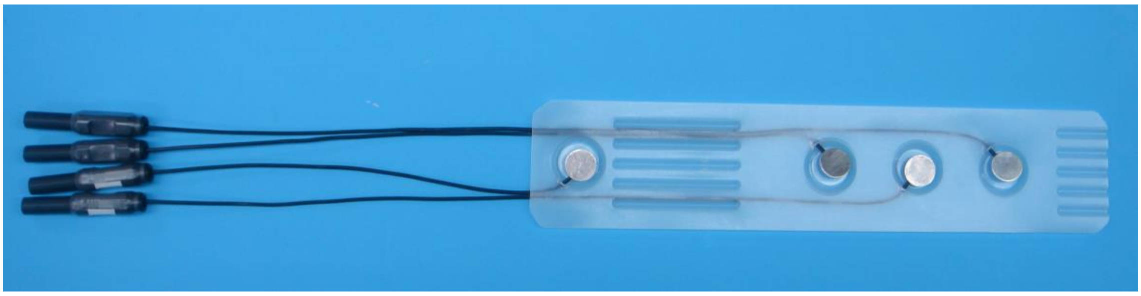

Complete view of the newly developed interface device used between commercially available needle electrodes and the existing BIS sensor

The left part consisting of four black electrodes enables to connect to NE-220B, the needle electrodes for EEG manufactured by Nihon Koden.

The right part is the conversion device receiving a commercially available BIS sensor, and the part with silver plate electrodes is reusable silver/silver chloride plate electrodes, which enabled to get the optimal electrical resistance through a test as diagramed below.

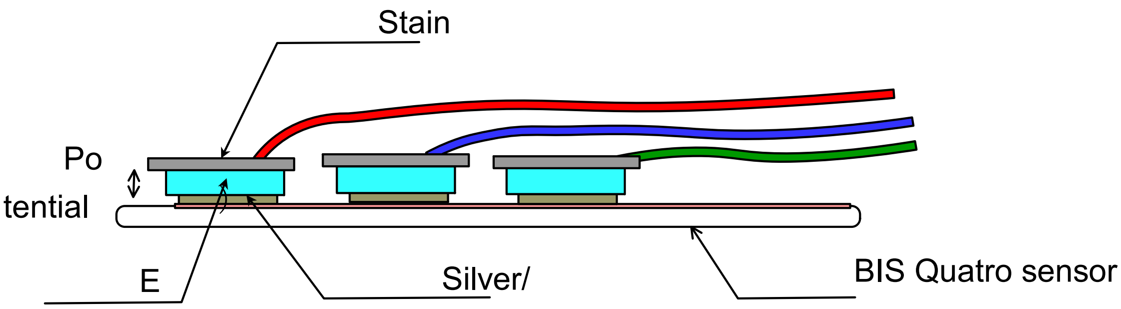

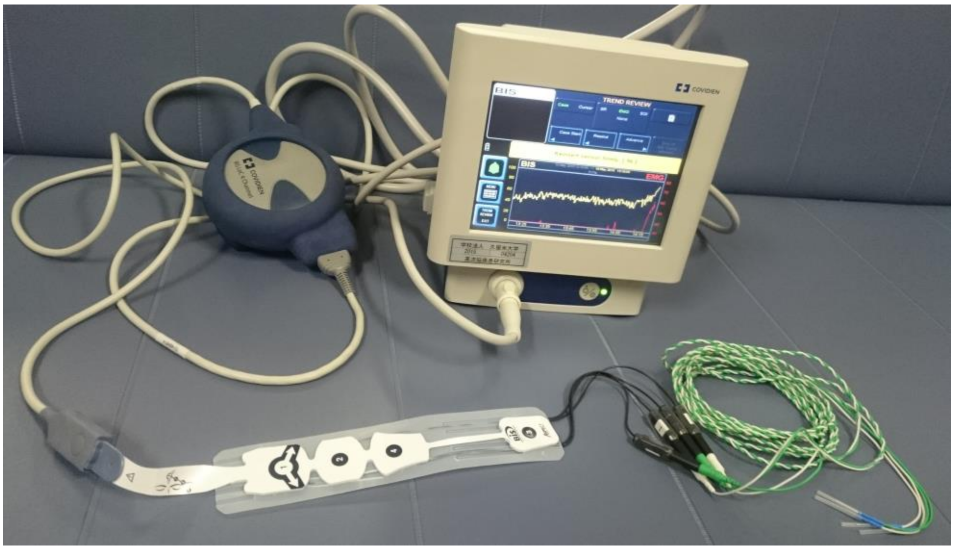

4. Aim of the study

To evaluate the clinical performance and safety of a newly developed BIS monitoring converting device using already existing needle electrodes in patients undergoing perioperative EEG monitoring as a subject.

5. Study design

Prospective study

6. Method of participant selection

6-1. Eligible patient and method of recruitment

Those who undergo surgery under intraoperative management by total intravenous anesthesia in the Department of Neurosurgery or Plastic Surgery at Kurume University Hospital

The target number of cases: 20 patients

6-2. Inclusion criteria

Patients who fulfill all of the following will be eligible:

・Patients aged 20 years or older when the consent was obtained
・Patents who provided written consent
・Patients with ASA1 or 2

6-3. Exclusion criteria

Patients who fulfill any of the following will be excluded:

・Patients with injury or skin disease on the forehead

・Patients with altered levels of consciousness before surgery due to intracranial disorders

・Patients with a surgical procedure impeding BIS sensor attachment at the forehead

・Patients with a neurological disorder

・Patients with a psychiatric disorder

・Patients with pathergy test positive

・Patients with metal hypersensitivity

7. Study period

Study period: from June, Heisei 28 (2016) after approval by the ethic committee until December 31, Heisei 30 (2018)

8. Study methods

In patients undergoing regular surgery in the Departments of Surgery or Plastic Surgery, who are planned for electrophysiological monitoring and provide consent, the study will be conducted as follows:

8-1. Detailed procedure

The patient will be brought into the operation room with usual medical procedure, and a commercially available BIS sensor will be attached to the forehead of the patients immediately after the introduction of anesthesia needle electrodes, and the perioperative consciousness levels and association in various BIS-related values will be compared.

I. Before the introduction of anesthesia, both needle electrodes and a commercially available BIS sensor will be attached at the same place of the forehead

II. Anesthesia will be introduced by propofol, remifentanil, and rocuronium

III. Normal anesthesia management will be done

IV. Because the needle electrodes and the commercially available BIS sensor are at the same position, there should be no difference. The BIS monitoring system used in this study can display BIS

values from two areas, and the electrodes are attached at the same position. The homology of the EEG wave form will be observed, values calculated using EEG processing, such as SQI, SEF, Beta-ratio, and resting ratio, will be compared and recorded if there is any discrepancy and if so in which degree.

V. After completion of the surgery, it will be recorded if there is any discrepancy and if so in which degree in increasing BIS values due to emergence.

8-2. Devices to be used (All of them are medical devices approved for clinical use)

1. NE-220B, the needle electrodes for EEG manufactured by Nihon Koden (length, 13mm; diameter 0.4mm).
2. BIS™ Complete 4 Channel Monitoring System
3. BIS™ Bilateral Sensor

8-3. Methods of allocation

This study acquires and compares information from two sensor locations in a single person. Therefore, there is no allocation.

8-4. Endpoints

Primary endpoints : BIS-related values from two different measurement locations

Secondary endpoints: perioperative complications

8-5. Analysis methods

By repeated ANOVA (post hoc test) and sequential two-group comparisons of BIS-related values, it will be proven that the observed values are the same.

9. Discontinuation criteria

At the following conditions, the study will be discontinued.

・Consent is withdrawn by patients

・Study physicians decide it inappropriate to continue the study

10. Correspondence to participants after conducting the study

After completing the study, we arrange participants being able to receive the best medical care (prevention, diagnosis, and treatment), obtained from the results of this study.

11. Expected benefits and disadvantages of participation

11-1. Expected benefits by participating in this study (effect)

Although the intraoperative anesthesia depth could be evaluated more strictly, the study subjects will not receive any direct benefit, because the study is observational, conducted within the scope of daily routine practice. Participants could contribute to future progress in medicine by providing the study results.

11-2. Expected disadvantages, adverse events, risks by participating in this study

The study uses a needle electrode NE-220B for EEG, manufactured by Nihon Koden, which has been clinically used for many years (length, 13mm; diameter 0.4mm). Symptoms observed by the use of original electrodes, such as subcutaneous bleeding and infection, may occur, but the occurrence is accidental and will be extremely rare.

12. Protection of the participants

12-1. Compliance with ethical standards

This study will be conducted in accordance with the ethical principles of the Declaration of Helsinki and the “Ethical Principles for Medical Research Involving Human Subjects”. To conduct this study, it should be approved by the ethic committee of the University of Kurume.

12-2. Methods for protecting personal information

Investigators being involved in the study execution take protection of privacy and personal information of participants well in consideration. The principal investigator will establish the necessary system to protect data and other information when conducting the study. If the data obtained in this study will be used for other purposes than this study, another consent will be obtained from the patient as necessary.

13. Consent of patients (informed consent)

Before perioperative period, consent will be obtained from the participants after explaining about the study. A withdrawal form will be handed out, and if the patient withdraws the consent by giving the withdrawal form or explaining verbally before the anesthesia introduction, the patient will be excluded from the study.

14. Costs burden to participants and compensation for loss

14-1. Costs burden

The study will be conducted within the scope of daily routine practice and the coverage of patient's health insurance. Therefore, observation, investigations, and used medications will be covered by the patient using the health insurance. The cost required for the use of needle electrodes and the newly developed BIS monitoring will be covered by the Department of Anesthesiology and there will be no additional cost for patients.

14-2. Loss compensation for health hazards

If health hazards occur due to this study, in principle, the treatment will be covered by the health insurance of the participant, and proper treatment and necessary measures will be taken. Because the cost will be borne by the patient, there will be no monetary compensation from the study institution.

15. Management and disposal methods for specimens and information (data)

Data regarding this study will be anonymized in a linkable fashion and be stored on a computer that belongs to the Department of Anesthesiology and is not connected to the internet. The signed consent forms and other forms will be stored in locked cabinets in the Department of Anesthesiology. Storage period is for five years. All documents related to the study except medical information will be shredded and disposed.

16. Funding source and conflict of interest

This study will be carried out using the internal research budget of the Department of Anesthesiology and will not be funded by external companies. Therefore, there is no conflict of interest.

17. Property of the results (patent)

Newly generated intellectual properties from this study will belong to the University of Kurume.

18. Disclosure of the study information and results

The results generated from the study will be presented at academic meetings of the Japanese Society of Anesthesiologists and the American Society of Anesthesiologists, and will be published in an academic journal.

19. Secondary use of data (ancillary studies)

It is possible to conduct ancillary studies using the data (specimens and information) from the study. In that case, the specimens and data will be anonymized and privacy will be protected. To conduct an ancillary study, the data will be used after a new study protocol is reviewed and approved by the ethic committee.

20. Contact

Harada Hideki, Department of Anesthesiology, University of Kurume School of Medicine. Ext. 3606
