# Supplementary material for: Accuracy of BIS monitoring using a novel interface device connecting conventional needle-electrodes and BIS sensors during frontal neurosurgical procedures
Source: PLoS One. 2021 Oct 21;16(10):e0258647. doi: 10.1371/journal.pone.0258647 (PMC8530286; doi:10.1371/journal.pone.0258647)
Supplement: Attachment — Submitted filename: Response to reviewers.docx [file pone.0258647.s006.docx]

Response to Reviewers

Dear Dr. Pasin,

Thank you very much for the review comments. We were very pleased to receive a chance to resubmit our manuscript and appreciate you and all reviewers for helping us to improve the manuscript. All comments were taken seriously, the manuscript was revised thoroughly, and we resubmit herewith our revised manuscript. In this letter, we describe our point-by-point responses to the reviewers.

We would appreciate very much for your consideration to publish our revised manuscript in PLOS ONE and hope the revised manuscript reached your criteria to accept it for publication.

Sincerely,

Miyuki Tauchi and Hideki Harada, on behalf of all coauthors

Point-by-point responses:

Journal Requirements:

1. Please ensure that your manuscript meets PLOS ONE's style requirements, including those for file naming. The PLOS ONE style templates can be found at

https://journals.plos.org/plosone/s/file?id=wjVg/PLOSOne_formatting_sample_main_body.pdf and

https://journals.plos.org/plosone/s/file?id=ba62/PLOSOne_formatting_sample_title_authors_affiliations.pdf

We apologize for your inconvenience. We paid attention to specific formatting during the revision so that the revised manuscript and the file names should be as required in PLOS ONE. The modifications are not tracked in the manuscript but the text has not been changed.

2. Thank you for submitting your clinical trial to PLOS ONE and for providing the name of the registry and the registration number. The information in the registry entry suggests that your trial was registered after patient recruitment began. PLOS ONE strongly encourages authors to register all trials before recruiting the first participant in a study.

As per the journal’s editorial policy, please include in the Methods section of your paper:
1) your reasons for your delay in registering this study (after enrolment of participants started);
2) confirmation that all related trials are registered by stating: “The authors confirm that all ongoing and related trials for this drug/intervention are registered”.

As you can see from the dates given in the manuscript, we started the recruitment of patients before the study was registered in a public domain. The reason was because of intellectual property for this device. Not like a medicinal product, device development largely depends on the idea. Because manufacturing of a device is not difficult and could be done very quickly, the idea can be easily stolen, once it is in a public domain. Accordingly, we consulted the Japanese ethic guidelines for an unapproved medical device in clinical studies; according to the guidelines, it is ethically allowed to register later in such a case, as long as it was approved by an ethic committee. Consequently, we started the patient recruitment before the registration, but of course after approval by an independent ethic committee. We registered the study after we ensured our intellectual property.

We added the reason for this discrepancy and stated in the method section of the manuscript that all currently ongoing, related trials are registered.

3. Please include captions for *all* your Supporting Information files at the end of your manuscript, and update any in-text citations to match accordingly. Please see our Supporting Information guidelines for more information: [http://journals.plos.org/plosone/s/supporting-information](about:blank).

We included a caption in the S1 file. We decided to withdraw the S2 (Please referrer our explanation below in the next paragraph).

4. We note that Figure [S2.BIS setting with the interface device (video)] includes an image of a patient in the study.

As per the PLOS ONE policy ([http://journals.plos.org/plosone/s/submission-guidelines#loc-human-subjects-research](about:blank)) on papers that include identifying, or potentially identifying, information, the individual(s) or parent(s)/guardian(s) must be informed of the terms of the PLOS open-access (CC-BY) license and provide specific permission for publication of these details under the terms of this license. Please download the [Consent Form for Publication in a PLOS Journal](about:blank) ([http://journals.plos.org/plosone/s/file?id=8ce6/plos-consent-form-english.pdf](about:blank)). The signed consent form should not be submitted with the manuscript, but should be securely filed in the individual's case notes. Please amend the methods section and ethics statement of the manuscript to explicitly state that the patient/participant has provided consent for publication: “The individual in this manuscript has given written informed consent (as outlined in PLOS consent form) to publish these case details”.

If you are unable to obtain consent from the subject of the photograph, you will need to remove the figure and any other textual identifying information or case descriptions for this individual.

We had obtained written consent from this patient and received permission to publish the data, including image, in a medical journal. However, it was not specifically for PLOS ONE or publicly available open-access journal. Considering that the patient is potentially identifiable in this video, we decided to withdraw it from the submitted materials. The photograph in Fig 3 was actually taken from the video. The photograph was further trimmed to make the individual absolutely unidentifiable.

5. Review Comments to the Author

Please use the space provided to explain your answers to the questions above. You may also include additional comments for the author, including concerns about dual publication, research ethics, or publication ethics. (Please upload your review as an attachment if it exceeds 20,000 characters)

Reviewer #1: Dear author, I congratulate for your work.
I have no comment. Your paper is appropriate and I believe room there is minimum space for further improvement.
I could only suggest to provide a better image 3B that appear to be blurry (too much light?)

Thank you very much for your positive comment. We are very encouraged.

Regarding the image B, we regret that we could not find another image with better quality. We tried to make it more understandable by adding an illustration.

Reviewer #2: In this manuscript, the authors demonstrated a novel interface device that connects conventional needle-electrodes to BIS monitoring sensors, thereby enabling the indirect BIS monitoring without topographical problems. This, otherwise, would be impossible to achieve using any of existing BIS sensors whose validation is largely relies on the use of it on the forehead. This interface device showed a good agreement between direct and indirect BIS values (2582/2787; 92.64%) within a clinically acceptable 10-unit difference. The concept and the experimental demonstration are interesting which I believe this manuscript is acceptable for publication in this journal if the followings can be addressed.

1. In this study, the authors insist that they developed the novel interface device, which connects the BIS Vista system to conventional EEG needle-electrodes for indirect BIS monitoring. I wonder that this interface device is novel enough. This interface system is constructed by connecting two components that are commercially available (BIS sensor and needle electrodes). In addition, the studies are already reported showing the use of needle electrode for BIS monitoring (T. M. Hemmerling et al., Anesth Analg, 2002, 95:1675-7, S. A. Greene et al., Comp. Med. 2002, 52:424-8). Thus, it is recommended for the authors to provide the novelty of this study compared to the previously reported works.

Thank you for your comment. We read the suggested articles carefully and discussed internally. As the result, we believe we can state our device novel.

As the reviewer points out, the idea to use needle electrodes is not novel. The above-mentioned literatures described briefly how they modified the commercially available sensors. Although the great details are missing (we also visited an original paper of Hemmerling et al, but there were no further details written), it seems that they handcrafted and modified the sensors (de-montage of parts, adhering wires, etc.), would be necessary to test the safety for each individual sensor, and the sensor would be probably one-way use. However, our device is an independent interface that connects two types of commercially available sensors without modifying them. This practical device offers a great advantage for practicing anesthetists.

The above given literature showed that the needle electrodes can be used interchangeably and thus support our study. We cited and discussed the literatures in our revised manuscript, highlighting the difference. Thank you very much again for introducing the literatures, which we had missed in the original version of the manuscript.

2. In Figure 5D’ and Figure 6C, even though SQI value was not very closely matched between indirect and direct recordings, the authors claim that their effect on the BIS correlation was minimal. Why is there little correlation? Besides, SQI value not for direct recording but for indirect recording was only strongly affected by automatic impedance check (Figure 5D). Why is the indirect recording only vulnerable to automatic impedance check? Are those recording results reliable with low value of SQI?

Before we go into the discussion, we would like to mention that it is not publicly available how the algorism calculates the BIS or SQI. During the course of study, we asked the Covidien representatives about this issue but could not get answer. We have been discussing why there is a discrepancy in SQIs and why the SQI spikes do not affect BIS very much. Upon the question from the reviewer, we went back to the raw data and discussed again. Our conclusion is that the SQI spikes are not because of the needle electrodes, nor because of the interface device, but because of the proprietary BIS algorism. We cannot give a firm answer, but our conclusion was drawn from our observation in raw data as follows:

When we see the raw data with all parameters in BIS recording (using Bilateral sensor in VISTA system), we can see that the impedance is measured at 4 electrodes individually (AVGIMPD 1-4). Interestingly, the values are 255 Ω exactly every 10 minutes for 1 (or occasionally 2) minute. This is obviously an artificial pattern: therefore, we consider that the 255 Ω is not an actual impedance value, but an artificial one because of the automatic impedance checks. Furthermore, this timing coincides with the SQI lowering. From this fact, SQI values are apparently dependent on AVGIMPD values.

To note, only 3 out of 4 electrodes display 255 Ω every 10 minutes (AVGIMP 2-4). AVGIMP 1 is not affected. Furthermore, SQI from the left side (in our experimental setting, dBIS recording) is based on AVGIMP 1 and 2, and that from the right side (indBIS recording) is based on AVGIMP 3 and 4. Thus, it is reasonable to think that the right side SQI based on AVGIMP 3 and 4 shows deeper spikes than the left side SQI. Thus, it is very likely not depending on the needle electrodes nor interface device.

We investigated and confirmed the followings: First, we took the raw data of BIS measurement, recorded by using the Bilateral sensor (no needle electrodes, no interface device), from several patients. We confirmed that, in all cases, AVGIMP 1 was not affected and AVGIMP 2-4 showed 255 Ω every 10 minutes. Then we plotted the SQI based on AVGIMP 1 and 2 (left) and SQI based on AVGIMP 3 and 4 (right) from these patients. The figure below is a representative SQI graph. The blue line shows the SQI from left side and the red line from the right side. There is a discrepancy between the two SQIs, just like indBIS vs. dBIS, although both recordings were done through conventional sensor (direct recording) in this case.

Next, we went back to the SQI data from needle electrode with the interface device only, being connected to Quatro sensor, which is the data from the clinical study 1. In this measurement, SQI is assumingly calculated by integrating all impedance values. In this measurement, the SQI spikes are always small (graph below, green line).

From above observation, we concluded that the observed SQI spikes are not needle- or interface device-specific. Confusingly, there are the Bilateral comparison data, which showed a very good match between dBIS and indBIS (below, top), but also larger spikes in dBIS (blue) than indBIS (red) (below, center). At the same time, there are also data from Bilateral sensor but both left and right sides were measured by conventional sensor, but left side (blue) has larger spikes (below, bottom).

As written earlier, how SQI is calculated is not publicly available. Therefore, we can only speculate from our available data and we regret that our answers to “why…” by the reviewer are not concrete. Nonetheless, we would like to stress that the SQI spikes do not substantially influence on the BIS values as a fact from the data.

Please do not publish the graphs above, as some of the graphs are obtained unrelated to this study and the written consent was not provided. Accordingly, we did not include this data in the manuscript. However, we explained our hypothesis regarding the SQI spikes much more in detail, which, we believe, sufficiently explains this issue.

3. By the way, there are typing errors:
- In page 21, line 393-394, EGM should be corrected to EMG.
- In Figure 7B, Y axis, “differece” should be corrected to “difference”.

Thank you for pointing them out. We corrected these errors and also reviewed again the manuscript thoroughly.

Reviewer #3: The paper was well presented. The data supported the conclusions. A minor revision is needed. The authors did not make the data fully accessible. Only the results of statistical analyses are available in the manuscript.

The raw data used for the analyses are available upon reasonable request. In our study, the participant number is small and the study sites locate in a small city, we feel the data can potentially identify the patient. Considering that, we decided not to make the data publicly available. However, upon reasonable request, we can examine if the requested data set bears any risk for identification and send the specific data sets after ensuring anonymity and approval from the ethic committee at the University of Kurume. At the end of the manuscript, we added a paragraph for the data availability and stated that we will share the data upon reasonable request.

Reviewer #4: The statistics are mainly regression with linear mixed models and the Bland Altman procedure for method comparison. These appear to be applied appropriately. However, there are major concerns.

1. The study lacks a reasonable statistical hypothesis for which the sample size of 18 to 21 should be justified in terms of the alpha level and anticipated power, even if the unit of analysis is otherwise. The entire design is not clear. Why so many points on Figure 7? This statistical justification should be provided.

Thank you for the comment. The main object of this study was to examine agreement between two methods. With regard to sample size and statistical power, formal power analysis was not conducted since there was no clear scientific and clinical guidelines for “equivalence margins” for agreement. To this end, this study was designed as exploratory, and we set BIS value differences of ±10 between dBIS and indBIS, which are clinically acceptable agreement for sake of interpreting results of data analyses. We added more detailed explanation of the design in the manuscript.

There are 2391 pairs of observations with BIS<60, and 395 pairs with BIS≥60. These pairs of observations were plotted in the figure 7. The plot is for all observation of patients throughout the measurement period. To make this clear, we added the number in the figure legend.

2. The Bland Altman procedure (Figure 7) is suspect and poorly explained. There is a great bulk of points beyond what appears to be the lower (+10, -10) bias limit. This limit is not very meaningful as presented with so many points violating those limits especially on the low side. This is not convincing for agreement. Also, what exactly are the 95% limits and how do they actually affect the overall conclusion?

Thank you for the comment. As written above, there are many measurement points in each patient. Because multiple pairs of observations were measured over various surgery periods within each patient, there were within-subject and between-subject variabilities. In order to account these two sources of variabilities in accessing agreement, we employed the methods suggested by the Parker RA et.al (2016), where 95% confidence intervals on the estimated BIS value differences obtained from the mixed-effects models were considered as Bland-Altman’s limit of agreement. This article is in the reference list #7.

3. Figure 6, A prime does not support the conclusion of the paper. There does not appear to be an acceptable level of agreement of the indirect and direct BIS. What is the hypothesis test of the slope and intercept on this line, especially the test for intercept equal to 0 and the slope equal to one?

Thank you for your comets. We would like to remind you that figure 6 is from a single person as a representative of an unstable BIS, because of artificial disturbance of EMG by neuromonitoring. We modified the manuscript so that the readers can easily recognize that figures 5 and 6 are just exemplary and from a single person for each. Figures B’, C’ and D’ are depiction of one patient’s observations with simple regression lines overlaid. R2 was shown as a measure of concordance between two paired measurements. No formal testing was performed on the estimates of intercept and slope. Of note, these data, although we believe the unstable BIS is an artefact because of neuromonitoring, are included in the formal testing described in the manuscript and plotted in Fig 7.
