# Supplementary material for: Accuracy of BIS monitoring using a novel interface device connecting conventional needle-electrodes and BIS sensors during frontal neurosurgical procedures
Source: PLoS One. 2021 Oct 21;16(10):e0258647. doi: 10.1371/journal.pone.0258647 (PMC8530286; doi:10.1371/journal.pone.0258647)
Supplement: Attachment — Submitted filename: point-by-point response.docx [file pone.0258647.s007.docx]

Dear Dr. Pasin,

Dear Editorial office,

Thank you very much for giving us another chance to discuss our manuscript. Here we provide a point-by-point response to the last review comment. We do not have any new changes in the manuscript. We appreciate in advance for your consideration.

The decision email and the review comments to the author are as follows:

**Decision email from the editor:**

Dear Dr. Harada,

Thank you for submitting your manuscript to PLOS ONE. After careful consideration, we have decided that your manuscript does not meet our criteria for publication and must therefore be rejected.

I apologize for the long time you had to wait. Unfortunately it was very, very difficult to find reviewers.

My decision is based on the lack of formal testing on the estimates of intercept and slope, as pointed out by Reviewer #4 (Point-by-point response 1).

I am sorry that we cannot be more positive on this occasion, but hope that you appreciate the reasons for this decision.

Yours sincerely,

Laura Pasin

Academic Editor

PLOS ONE

**Reviewers' comments:**

*6. Review Comments to the Author*

*Please use the space provided to explain your answers to the questions above. You may also include additional comments for the author, including concerns about dual publication, research ethics, or publication ethics. (Please upload your review as an attachment if it exceeds 20,000 characters)*

Reviewer #4: With respect to the BIS regressions, the reviewer asked:

What is the hypothesis test of the slope and intercept on this line, especially the test for intercept equal to 0 and the slope equal to one?

The authors responded:

No formal testing was performed on the estimates of intercept and slope. Of note, these data, although we believe the unstable BIS is an artefact because of neuromonitoring, are included in the formal testing described in the manuscript and plotted in Fig 7 (Point-by-point response 2).

Such may be the case. However, an R-square less than 0.9 when considering agreement is not that convincing. At best, the agreement is weak, statistically (Point-by-point response 3).

In Figure 7A you want the intercept to be 0 and the slope to be one (Point-by-point response 4). A formal test not rejecting these null hypotheses that intercept=0 and slope=1 would convince one of agreement. That was the reason for requesting a formal test (Point-by-point response 5).

**(****Point-by-point response 1) The formal testing for single-person’s data is not relevant**

Here the editor wrote that her decision was based on the lack of formal testing on the estimates of intercept and slope, as pointed out by reviewer 4. We would like to remind you about our response to the first review: The data plots and their regression line, for which the reviewer 4 asked about the hypothesis test, are based on a single patient. “Formal testing result” based on a single person’s data cannot be interpreted. Otherwise, there is no reason to select this individual to do the formal test and generalize the result. Therefore, we did not perform the formal test for this particular case. In the manuscript revised previously, we had modified the text to state clearly that the data are based on a single person (L234 and L257 in the “Revised Manuscript with Track Changes.docx”).

We would also like to stress that we did two formal testing to interpret the data. Please refer the point-by-point response 5 below.

**(****Point-by-point response 2) In this comment, only selected parts of reviewer’s question and our response are taken, resulted in a misleading sentence.**

Please refer the complete question and response below. The parts the reviewer chose for his/her comment are highlighted in blue.

*3. Figure 6, A prime does not support the conclusion of the paper. There does not appear to be an acceptable level of agreement of the indirect and direct BIS. What is the hypothesis test of the slope and intercept on this line, especially the test for intercept equal to 0 and the slope equal to one?*

Thank you for your comments. We would like to remind you that figure 6 is from a single person as a representative of an unstable BIS, because of artificial disturbance of EMG by neuromonitoring. We modified the manuscript so that the readers can easily recognize that figures 5 and 6 are just exemplary and from a single person for each. Figures B’, C’ and D’ are depiction of one patient’s observations with simple regression lines overlaid. R2 was shown as a measure of concordance between two paired measurements. No formal testing was performed on the estimates of intercept and slope. Of note, these data, although we believe the unstable BIS is an artefact because of neuromonitoring, are included in the formal testing described in the manuscript and plotted in Fig 7.

The reviewer was asking which statistic we did for this specific case (Figure 6, highlighted in green). Therefore, we responded that we did not perform any formal test for this particular data set and provided explanation why (highlighted in gray). In any case, we took the comment seriously and interpreted that the nature of the data was not written clearly enough. Therefore, we modified the manuscript to avoid this misunderstanding (underlined and tracked at L234 and L257 in the “Revised Manuscript with Track Changes.docx”).

However, in the second response, the comment-response text is truncated and used only the part marked in blue. This version of comment-response is misleading and gives an impression that we did not perform formal testing at all despite a request from the reviewer, which is not true. We would like kindly to ask to read the entire comment-response text for the evaluation.

**(****Point-by-point response 3) As written earlier, the data is from a single person and therefore no formal testing is relevant.**

As written in the point-by-point response 2, the data in Fig. 6 were from **a single patient**, as an example of an unstable case, and **therefore no formal testing was performed**. We did not do so, because “formal testing result” based on a single person’s data cannot be interpreted. Accordingly, we do not discuss about any statistically weak or strong correlation. The simple regression line was overlaid and r^2^ was calculated merely to obtain the tendency within the individual patient.

We absolutely agree that we have to perform formal statistical testing to interpret the (entire) data. To do so, we performed two types of formal testing using the entire data set from 18 patients. Please refer point-by-point response 4.

**(****Point-by-point response 4) No. We did not want to test a simple linear regression. Instead, we did two different types of formal testing, which we consider appropriate.**

The reviewer wrote “In Figure 7A you want the intercept to be 0 and the slope to be one”. No, we did not want to perform the test the reviewer 4 suggested, but two other testing. There are reasons why we took different approaches.

First, the number of data points from individual patient differs. In our experiments, some patients underwent a short surgery, and others a long surgery. Consequently, some people contributed to only a small proportion of the data set, and others much larger. The difference in the number of data points in each individual has to be correctly handled.

Another reason is that the data were repeatedly sampled from each patient during his/her surgery. Therefore, the data are not independent, which has to be considered in the statistical model.

With the reasons above, we took different approaches for the formal testing.

**(****Point-by-point response 5) We had performed two powerful and statistically sound testing, which are appropriate for the type of data we have.**

The reviewer wrote “A formal test not rejecting these null hypotheses that intercept=0 and slope=1 would convince one of agreement”. We disagree. “Not able to reject null hypothesis A=B” is NOT equal to “therefore A=B”. It simply means, “A≠B cannot be concluded”.

To demonstrate the equivalence, an appropriate “equivalence test” should be used with known equivalence margins that are supported by scientific evidence. In our case, however, no such margins exist. This is why we did not perform an equivalence test but did two other types of formal testing instead, which were appropriate for this type of data. First, we tested the concordance of the values using the linear mixed model and demonstrated significant concordance. In addition, we performed a test using the modified BA plot, as stated in the former paragraph. Please see the statistics section of the manuscript and the supplementary text, where we described the methods in detail. These tests we performed were powerful and scientifically sound.

Here we submit the rebuttal against the review comment. We do not have any changes in the manuscript this time. We cordially ask you for a careful evaluation.

Thank you very much in advance.

Sincerely,

Dr. Miyuki Tauchi, Laboratory for molecular Cardiology, University Hospital Erlangen, Germany

Prof. Hideki Harada, Cognitive and molecular institute of Brain Diseases Kurume University, Japan

Prof. Tatsuyuki Kakuma, The Biostatistics Center, Kurume University, Japan

On behalf of all other coauthors
